# Protein arginine methyltransferases: promising targets for cancer therapy

**DOI:** 10.1038/s12276-021-00613-y

**Published:** 2021-05-18

**Authors:** Jee Won Hwang, Yena Cho, Gyu-Un Bae, Su-Nam Kim, Yong Kee Kim

**Affiliations:** 1 Research Institute of Pharmaceutical Sciences, College of Pharmacy, Sookmyung Women’s University, Seoul, 04310 Republic of Korea; 2 Natural Product Research Institute, Korea Institute of Science and Technology, Gangneung, 25451 Republic of Korea

**Keywords:** Drug development, Methylation

## Abstract

Protein methylation, a post-translational modification (PTM), is observed in a wide variety of cell types from prokaryotes to eukaryotes. With recent and rapid advancements in epigenetic research, the importance of protein methylation has been highlighted. The methylation of histone proteins that contributes to the epigenetic histone code is not only dynamic but is also finely controlled by histone methyltransferases and demethylases, which are essential for the transcriptional regulation of genes. In addition, many nonhistone proteins are methylated, and these modifications govern a variety of cellular functions, including RNA processing, translation, signal transduction, DNA damage response, and the cell cycle. Recently, the importance of protein arginine methylation, especially in cell cycle regulation and DNA repair processes, has been noted. Since the dysregulation of protein arginine methylation is closely associated with cancer development, protein arginine methyltransferases (PRMTs) have garnered significant interest as novel targets for anticancer drug development. Indeed, several PRMT inhibitors are in phase 1/2 clinical trials. In this review, we discuss the biological functions of PRMTs in cancer and the current development status of PRMT inhibitors in cancer therapy.

## Cancer therapy: Exploring possible enzyme targets

Understanding the roles, regulation and downstream targets of a family of enzymes involved in cancer development could improve future therapies. Yong Kee Kim at Sookmyung Women’s University, Seoul, South Korea, and co-workers reviewed current knowledge of protein arginine methyltransferases (PRMTs), their influence on cell cycle regulation and DNA damage responses in cancer, and their potential as anti-cancer targets. PRMTs act as catalysts in modification of the amino acid arginine. This process is critical to healthy cellular function, and its disruption is linked to cancer development. The overexpression of certain PRMTs is a driving factor in cancer tumor growth, metastasis and poor prognosis. PRMT inhibitors are showing promise in clinical trials for B-cell lymphoma and various solid tumors. Combined therapies targeting more than one PRMT could prove valuable.

## Introduction

**Fig. 1:**
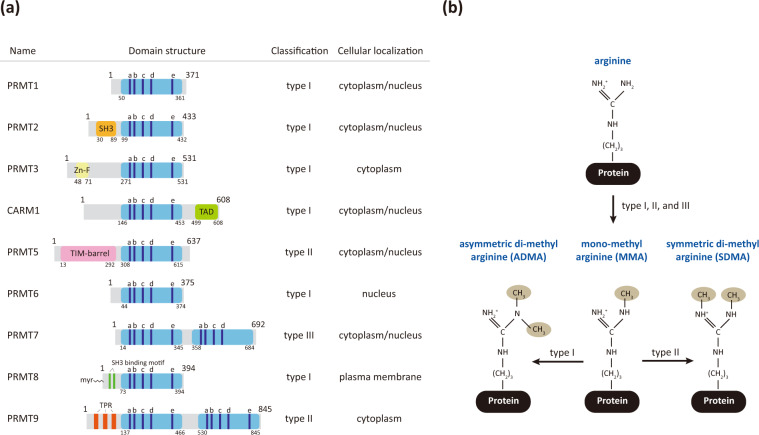
Protein arginine methylation and responsible enzymes. **a** The mammalian PRMT family. Nine PRMTs were identified, and these have unique signatures (dark blue lines) with high sequence similarity (a, Motif I: VLD/EVGXGXG; b, Post-I: V/IXG/AXD/E; c, Motif II: F/I/VDI/L/K; d, Motif III: LR/KXXG; e, THW loop). Their enzymatic types and cellular localization are shown. **b** Types of arginine methylation. The arginine residue has two equivalent nitrogen atoms in its guanidino group. Types I, II, and III PRMTs generate monomethyl arginine (MMA) marks. The subsequent generation of asymmetric dimethyl arginine (ADMA) is catalyzed by type I enzymes (PRMT1, PRMT2, PRMT3, CARM1, PRMT6, and PRMT8), and symmetric dimethyl arginine (SDMA) is produced by type II enzymes (PRMT5 and PRMT9). PRMT7, a type III enzyme, generates only MMA.

Since the discovery of arginine residue methylation on histone proteins^1^, protein arginine methylation has been emphasized as an indispensable post-translational modification (PTM) and an epigenetic regulation mechanism^2,3^. Arginine methylation is catalyzed by a family of enzymes called protein arginine methyltransferases (PRMTs), and nine PRMTs have been identified in mammals to date (Fig. 1a)^2,4,5^. All PRMTs share four conserved sequence motifs (I, post-I, II, and III) and one THW loop, which compose the S-adenosyl-L-methionine (AdoMet) binding pocket in the tertiary structure^6,7^. PRMTs transfer a methyl group from the AdoMet molecule to the guanidino group of the arginine residue in substrate proteins^8^. There are three types of methyl arginine (Fig. 1b): ω-*N*^*G*^-monomethyl arginine (MMA), ω-*N*^*G*^*,N*^*G*^-asymmetric dimethyl arginine (ADMA), and ω-*N*^*G*^*,N’*^*G*^-symmetric dimethyl arginine (SDMA)^8^. PRMTs are classified into three subgroups based on the type of methyl arginine they produce: Type I PRMTs (PRMT1, 2, 3, 4, 6, and 8) generate MMA and ADMA, Type II PRMTs (PRMT5 and 9) produce MMA and SDMA, and Type III PRMT (PRMT7) produces only MMA^7,9^.

**Fig. 2:**
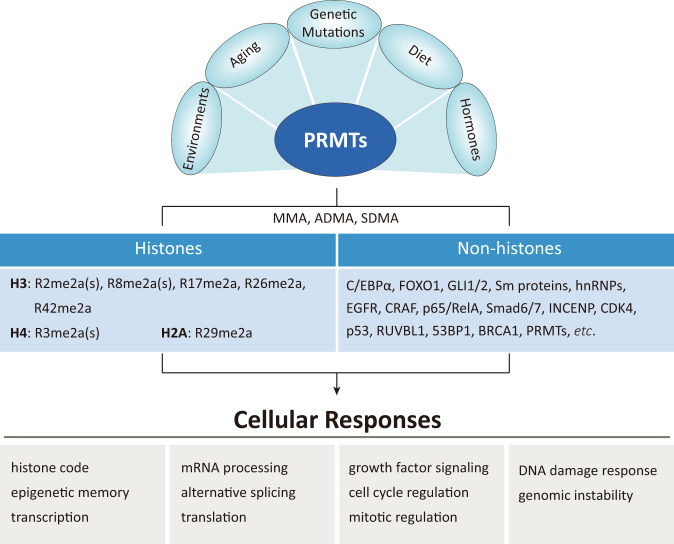
Biological functions of protein arginine methylation. Protein arginine methylation is observed in both histones and nonhistone proteins, which contribute to diverse cellular responses for maintaining cellular homeostasis in biological systems. The expression and activity of PRMTs are regulated by developmental and pathogenic processes, genetic mutations, and various environmental factors.

The arginine residue consists of a guanidino group on its side chain, which is protonated and positively charged at physiological pH^3,5^. The guanidino group forms multiple hydrogen bonds that bind with other interacting proteins or cofactors^2,5^. Although the methylated arginine residue retains its positive charge, the ability to form hydrogen bonds is reduced, probably affecting the protein-protein interaction. In addition, arginine methylation is very stable compared to that of other PTMs, and hence, its kinetics are less dynamic^2,5^. PRMTs are associated with many essential cellular processes, including transcription, splicing, translation, signal transduction, DNA damage and repair, and cell cycle regulation (Fig. 2)^2–4^, and the knockout phenotypes of some PRMTs show embryonic or perinatal lethality^2,10–12^, indicating the significance of PRMTs in maintaining functional homeostasis in biological systems. Tissue-specific deletion studies of PRMTs strongly support the supposition that they are involved in cancer and metabolic, immune, neurodegenerative, and muscular disorders^4,13,14^. Since the dysregulation of PRMTs has been closely associated with cancer development^2,15,16^, the use of PRMTs as novel targets for anticancer drug development is rapidly increasing. Recent studies have revealed considerable advances in the identification of clinically relevant PRMT inhibitors^17,18^. Here, we focus on the biological functions of PRMTs in cancer and the therapeutic potential of PRMT inhibitors.

## Biological functions of protein arginine methylation

As histone proteins tightly regulate gene transcription through various PTMs, including acetylation, lysine methylation, phosphorylation, ubiquitination, and SUMOylation^19,20^, early studies of PRMTs have also focused on their epigenetic functions. PRMTs synthesize methyl arginine on nucleosomes after being recruited into chromatin remodeling complexes, and these methylated arginine residues not only serve as key epigenetic marks but also engage in crosstalk with other epigenetic marks^21,22^. These orchestrated epigenetic modifications are recognized by epigenetic reader proteins, leading to the recruitment of activating/repressing transcriptional machinery. The histone modifications generated by PRMTs and their roles are summarized in Table 1. The methylation status of an arginine residue in histones can determine whether the transcription process is activated or suppressed. For example, H4R3me2a, a modification generated by PRMT1/PRMT3, acts as a mark of activated transcription, whereas H4R3me2s, generated by PRMT5, functions as a repression mark, implying that there is a sophisticated and competitive mechanism between PRMTs for regulating the transcription process. In addition to histone proteins, various proteins involved in transcription, such as transcription factors, coactivators, and corepressors, are also methylated by PRMTs (Table 1 and Fig. 2)^23^. Hence, PRMTs also contribute to the precise regulation of the transcription process. A number of RNA-binding proteins (RBPs) have RG/RGG-rich motifs that have been established as representative consensus sequences of PRMTs^24,25^. Indeed, theoretical insights and proteomic analysis revealed that several RBPs are methylated by PRMTs and that these modifications are essential for mRNA splicing, RNA localization, and translation processes^26–28^. In addition to gene expression regulation, the functions of PRMTs are extended to various cellular processes, including cell signaling, cell cycle regulation, and the DNA damage response (DDR)^2,3^. Methylation of arginine residues in signal receptors and their downstream mediators determines the amplitude or duration of signal transduction, contributing to the regulation of cell proliferation, survival, differentiation, and metabolism. Although all of these functions are critical for maintaining cellular homeostasis and normal cell growth, we highlight the biological roles of PRMTs in both cell cycle regulation and the DDR, which are the key pathways that are dysregulated in the hallmarks of cancer.

**Table 1:** The biological roles of PRMTs.

Substrate	Residues	Enzymes	Function	Ref
*Transcription-histone methylation*				
H4	R3me2a	PRMT1	Transcription activation	^140,141^
		PRMT3	Transcription activation	^142^
	R3me2s	PRMT5	Transcription repression	^143–145^
H3	R2me2a	PRMT6	Transcription repression	^146,147^
	R2me2s	PRMT5	Transcription activation	^79^
	R8me2a	PRMT2	Transcription activation	^148^
	R8me2s	PRMT5	Transcription repression	^97,149^
	R17/R26me2a	CARM1	Transcription activation	^150–152^
	R42me2a	CARM1/PRMT6	Transcription activation	^153^
H2A	R29me2a	PRMT6	Transcription repression	^154^
*Transcription–transcription factors*				
STAT1	R31	PRMT1	Activates STAT1 transactivity	^155^
C/EBPα	R35/156/165	PRMT1	Blocks the interaction with its corepressor, HDAC3	^156^
RUNX1	R206/210	PRMT1	Interferes with binding to SIN3A	^157^
FOXO1	R248/250	PRMT1	Stabilizes the FOXO1 protein	^158^
MyoD	R121	PRMT1	Increases MyoD transactivity	^159^
Nrf2	R437	PRMT1	Increases DNA-binding affinity and transactivity	^160^
Twist1	R34	PRMT1	Facilitates repressive activity at the *E-cadherin* promoter	^68^
p65/RelA	R30	PRMT1	Inhibits its own DNA-binding affinity	^161^
GLI1	R597	PRMT1	Enhances the recruitment of GLI1 to target gene promoters	^67^
CBP/p300	R714/742/768/2104/2151	CARM1	Enhances the HAT activity of CBP/p300	^162–165^
Sox2	R113	CARM1	Increases Sox2 transactivity	^166^
FoxO3	—	CARM1	Increases FoxO3 transactivity	^167^
Sox9	—	CARM1	Disrupts the binding between Sox9 and β-catenin	^168^
MED12	R1862/1912	CARM1	Suppresses *p21*^*WAF1*^ transcription	^75^
p65/RelA	R30	PRMT5	Enhances the transactivation of NF-κB	^169^
p64/RelA	R174	PRMT5	Increases TNF-α/IFN-γ-induced *CXCL11* gene expression	^170^
GLI1	R990/1018	PRMT5	Promotes proteasome-dependent degradation of GLI1	^171^
HOXA9	R140	PRMT5	Increases transactivation of HOXA9 in the *E-selectin* promoter	^172^
GLI2	R225/227	PRMT7	Dissociates GLI2 from SUFU	^173^
*mRNA splicing/alternative splicing*				
Sm D1, D3, B/B'	SDMA	PRMT5	Enhances binding with SMN	^26,174–176^
SmB/B’	ADMA	CARM1	Unknown	^177,178^
CA150		CARM1	Enhances binding with SMN	^178^
LSm4		PRMT5	Promotes interaction with HAT1-RBBP7	^179,180^
Coilin		PRMT5	Mediates SMN localization in the Cajal body	^28,181^
fibrillarin		PRMT1	Facilitates interaction with SMN	^182^
GAR1		PRMT1	Facilitates interaction with SMN	^182^
hnRNP A2		PRMT1	Regulates cytosolic/nucleus localization	^183^
hnRNAP Q		PRMT1	Regulates cytosolic/nucleus localization	^184^
hnRNP K		PRMT1	Promotes the interaction with c-Src	^185^
RBM15	R578	PRMT1	Promotes ubiquitination by E3 ligase CNOT4	^186^
KSRP		CARM1	Enhances interaction with SMN	^187^
ZNF326	R175	PRMT5	Regulates alternative splicing process	^188^
SAP145	R508	PRMT9	Promotes interaction with SMN and U2 snRNP maturation	^189,190^
*Translation*				
AVEN		PRMT1	Regulates translation in G-quadruplexes harboring mRNA	^191^
TOP3B	R833/835	PRMT1/3/6	Localizes to stress granules	^192^
rpS3	R64/65/67	PRMT1	Promotes ribosome assembly	^193^
rpS2		PRMT3	Inhibits ubiquitin-dependent degradation of rpS2	^194,195^
PABP1		CARM1	Unknown	^196^
hnRNP A1	R218	PRMT5	Controls IRES-dependent translation	^197^
RPS10	R158/160	PRMT5	Regulates the assembly of ribosomes	^198^
eIF2a	R54	PRMT7	Regulates stress granule formation	^199^
*Cell signaling*				
EGFR	R1175	PRMT5	Promotes association with SHP1 phosphatase	^200^
	R198/200	PRMT1	Increases binding affinity for EGF leading to dimerization of EGFR	^66^
CRAF	R563	PRMT5	Regulates degradation of CRAF	^201^
	R100	PRMT6	Regulates the binding affinity for RAS	^202^
p38 MAPK	R70	PRMT7	Enhances p38 MAPK activation	^203^
ASK1	R78/80	PRMT1	Promotes the association with thioredoxin	^204^
	R89	PRMT5	Promotes AKT-mediated Ser83 phosphorylation of ASK1	^205^
Smad6	R74	PRMT1	Facilitates the dissociation of Smad6 from type I receptors	^135^
Smad7	R57/67	PRMT1	Facilitates the dissociation of Smad7 from type I receptors	^134^
*Cell cycle*				
CDK4	R55/73/82/163	PRMT1	Inhibits CDK-Cyclin D3 complex formation	^31^
INCENP	R887	PRMT1	Facilitates interaction with AURKB	^32^
UBAP2L	RGG/RG motif	PRMT1	Promotes alignment of chromosomes in metaphase	^33^
H3	R2me2a	PRMT6	Recruits AURKB/CPC to chromosome arm during mitosis	^43^
*DNA damage response*				
MRE11	GAR motif	PRMT1	Activates exonuclease activity and recruits factors to damaged DNA	^44,45^
53BP1	GAR motif	PRMT1	Increases DNA-binding affinity	^47,48^
		PRMT5	Stabilizes the 53BP1 protein	^63^
DNA polymerase β	R137	PRMT1	Interferes with binding with PCNA	^50^
FEN1	R192	PRMT1	Interaction with PCNA and localization to damaged DNA foci	^51^
Rad9	R172/174/175	PRMT5	Activation of CHK1 signaling	^55^
p53	R333/335/337	PRMT5	Regulation of promoter selectivity	^57,58^
E2F1	R111/113	PRMT5	Downregulation of E2F1 protein stability	^59,206^
KLF4	R374/376/377	PRMT5	Inhibition of VHL-mediated ubiquitination	^60^
RUVBL1	R205	PRMT5	Increase in TIP60-dependent chromosome acetylation	^61^
p300/CBP	R754	CARM1	Recognition by BRCA1 and *p21*^*WAF1*^ induction	^54^

### Regulation of the cell cycle through arginine methylation

**Fig. 3:**
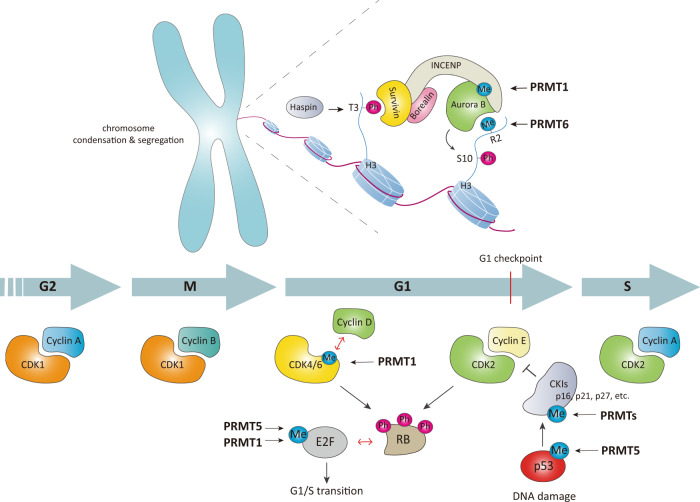
Regulation of the cell cycle through protein arginine methylation. The cell cycle is mainly regulated by phase-specific oscillation of cyclin-dependent kinase (CDK)-cyclin complexes. The expression of several cyclins (Cyclin E, Cyclin D1, etc.) and CDKs is epigenetically regulated by PRMTs (not shown). CDK4 is directly methylated by PRMT1, which inhibits binding with Cyclin D and blocks cell cycle progression. In contrast, methylation of E2F1 by either PRMT1 or PRMT5 results in cell progression from G1 to S phase. Several CKIs (CDK inhibitors), such as p16, p21, and p27, are directly methylated by PRMTs to regulate their binding with CDK-cyclin complexes or their cellular localization. During mitosis, PRMT6-mediated H3R2me2a recruits Aurora B kinase into chromosomes along with CPC components, enabling H3S10 phosphorylation. Another CPC component, INCENP, is also methylated by PRMT1, which promotes its interaction with Aurora B kinase. Together, the activities of PRMT1 and PRMT6 during M phase are required for chromosome condensation and proper segregation.

Cell cycle progression is precisely orchestrated by the cooperation of various signaling pathways and post-translational modifications^29,30^. Arginine methylation is implicated in the cell cycle via gene expression regulation or the direct methylation of cell-cycle-related regulators (Fig. 3).

PRMT1 methylates cyclin-dependent kinase 4 (CDK4), a key regulator of the G1-S transition, at four residues (Arg55/73/82/163) located near the Cyclin D3-binding area^31^. These multiple arginine methylations disrupt the formation of the CDK4-Cyclin D3 complex and advance cell cycle progression, promoting pre-B-cell differentiation and inhibiting leukemogenesis. The inner centromere protein (INCENP), a component of the chromosomal passenger complex (CPC), is methylated by PRMT1 at the Arg887 residue located in the Aurora kinase B (AURKB)-binding region^32^. The methylation of Arg887 in INCENP facilitates its interaction with AURKB, thereby augmenting AURKB activity and contributing to the enhancement of chromosome alignment and segregation during mitosis in cancer cells. PRMT1 also regulates chromosome alignment via arginine methylation of ubiquitin-associated protein 2-like (UBAP2L)^33^. PRMT1 directly interacts with and methylates UBAP2L on its N-terminal RGG/RG motif, and its methylation is essential for the proper alignment and accurate distribution of chromosomes in metaphase.

As a transcriptional coactivator, CARM1 (coactivator-associated arginine methyltransferase 1, also known as PRMT4) participates in cell cycle progression by regulating the expression of genes associated with the cell cycle. With the p160 coactivator member ACTR/SRC3/AIB1, CARM1 acts as a coactivator of Cyclin E (*CCNE1*) transcription in an E2F1-dependent manner^34^. CARM1 recruited to the *CCNE1* promoter increases the levels of H3R17me2a and H3R26me2a, resulting in transcriptional activation of *CCNE1* and subsequent cellular entry into S phase. *E2F1* is a target for transcriptional regulation of CARM1 for cell cycle regulation^35^. Upon estrogen stimulation, CARM1 is recruited to the *E2F1* promoter with ERα in an oncogenic coactivator AIB1-dependent manner and then induces the H3R17me2a modification at the *E2F1* promoter. The epigenetic upregulation of *CCNE1* and *E2F1* mediated by CARM1 is associated with the development of breast cancer.

PRMT5 activity is primarily implicated in G1 progression and the G1-S transition. PRMT5 overexpression accelerates cell cycle progression by increasing the expression of cell cycle regulators, including CDK4, CDK6, Cyclin D1, Cyclin D2, Cyclin E1, and phospho-Rb^36^. In addition, upregulation of PRMT5 activates PI3K, AKT, mTOR/eIF4E, and NF-κB signaling, contributing to the proliferation of cancer cells^36^. PRMT5 epigenetically suppresses *RBL2*, a member of the retinoblastoma tumor suppressor family, and indirectly enhances RB1 phosphorylation, resulting in the activation of the polycomb repressor complex PRC2 and Cyclin D1^37^. The upregulation of the expression of PRC2 and Cyclin D1 facilitates cell cycle progression and cell survival via activation of cyclin D1-CDK4/6 signaling and suppression of proapoptotic target genes of PRC2. As an alternative mechanism for Cyclin D1 upregulation mediated by PRMT5, the regulation of tumor suppressor miRNA expression by PRMT5 was recently studied, and the results were published^38^. PRMT5 epigenetically suppresses the expression of several tumor suppressor miRNAs, such as miR33b, miR96, and miR503, which bind to and target the mRNA corresponding to Cyclin D1 and/or c-Myc. In aggressive B-cell lymphoma, upregulated expression of PRMT5 leads to a decrease in the levels of these miRNAs and an increase in Cyclin D1 and c-Myc expression. PRMT5 directly interacts with CDK4, impeding the competitive interaction between CDK4 and p16^39^. This PRMT5-CDK4 complex promotes the activation of CDK4-pRb-E2F-mediated transcription and, in turn, the cell cycle progression of hepatocarcinoma cells.

PRMT6 regulates the cell cycle via epigenetic repression of cell cycle-related regulators, such as *p21*^*WAF1*^, *p27*^*KIP1*^, and *p18*^40–42^. The H3R2me2a modification mediated by PRMT6 transcriptionally turns off these genes and, in turn, induces abnormal bypass of the cell cycle. This outcome demonstrates the oncogenic function of PRMT6. The PRMT6-mediated H3R2me2a modification is essential for the recruitment of CPC to chromosome arms during mitosis^43^. AURKB preferentially binds to H3R2me2a and phosphorylates H3S10, which recruits the CPC complex to chromosome arms for precise chromosome condensation and segregation during mitosis.

### Regulation of the DNA damage response through arginine methylation

One of the important biological roles of arginine methylation is the regulation of DNA damage signaling and DNA repair processes. Several DDR regulators have been identified as substrates for PRMTs, and their methylated arginine residues modulate their functions, stability, DNA-binding affinity, and interaction with other proteins (Table 1).

The double-strand break repair protein MRE11, a component of the MRE11–RAD50-NBS1 (MRN) complex, is methylated by PRMT1 in its C-terminal glycine-arginine rich (GAR) domain^44,45^, which is critical for its exonuclease activity and allows the recruitment of MRE11 to the damaged DNA site^44^. PRMT1-mediated MRE11 methylation is implicated in the S-phase DNA damage checkpoint, ATR/CHK1 signaling, and the recruitment of replication protein A (RPA) and RAD51 to DNA lesions^45,46^, suggesting that arginine methylation in the GAR domain is required for the normal functioning of MRE11 in response to DNA damage stress and repair signaling. PRMT1 methylates arginine residues in the GAR motif in 53BP1 (p53-binding protein 1), a key regulator of the nonhomologous end-joining (NHEJ) repair process^47,48^. Asymmetrically dimethylated 53BP1 enhances DNA-binding activity without affecting its oligomerization. During the repair of DNA single-strand breaks and single-base lesions, DNA polymerase β (pol β) plays an indispensable role in the DNA base excision repair (BER) pathway^49^. PRMT1 methylates the Arg137 residue of pol β, which interferes with the binding of proliferating cell nuclear antigen (PCNA) without affecting its polymerase or dRP-lyase activities^50^. Another DNA repair regulator, Flap endonuclease 1 (FEN1), is methylated by PRMT1 at Arg192^51^. This methylation suppresses its phosphorylation at Ser187, promoting its interaction with PCNA and its localization to damaged DNA foci. Furthermore, upregulation of PRMT1 correlates with high expression of FEN1 in lung cancer due to stabilization of the FEN1 protein via PRMT1-mediated arginine methylation^52^.

The tumor suppressor BRCA1, a key regulator of the HR repair process, is directly or indirectly regulated by arginine methylation. PRMT1 interacts with and methylates the 504–802 region of BRCA1 to consolidate its target promoters^53^. The methylation status of the 504–802 region determines the binding preference of BRCA1 for SP1 or STAT1. Furthermore, methylation of the Arg754 residue of CBP/p300 by CARM1 is preferentially recognized by the BRCT domain of BRCA1, which is critical for the recruitment of BRCA1 to the p53-binding region of the *p21*^*WAF1*^ promoter^54^.

**Fig. 4:**
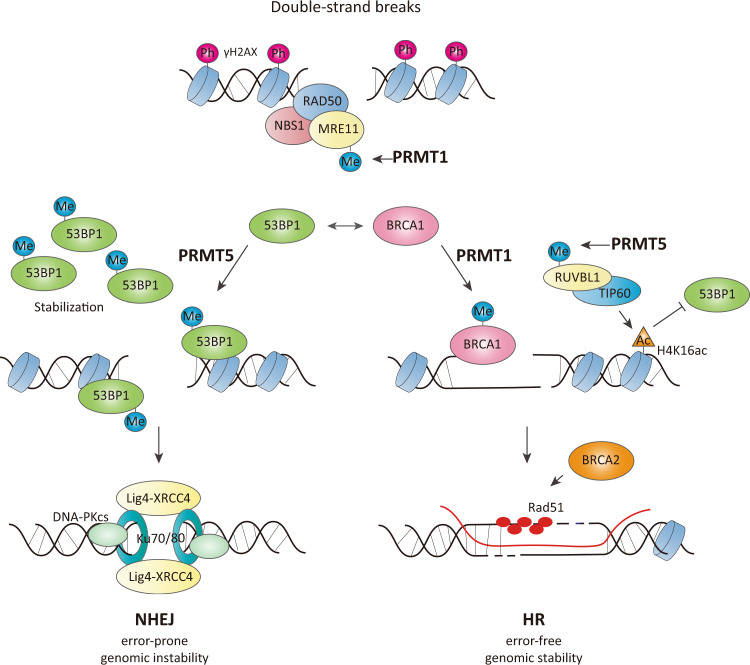
Regulation of the DNA damage response through protein arginine methylation. Under DNA double-strand breaks, the MRE11–RAD50–NBS1 complex is recruited into the DNA lesion and activates ATM/CHK2 kinase signaling. PRMT1-mediated MRE11 methylation is essential for exonuclease activity and localization to DNA. There are two main repair pathways, homologous recombination (HR) and nonhomologous end-joining (NHEJ). 53BP1, a major regulator of NHEJ, is competitively methylated by PRMT1 and PRMT5 in the GAR motif. PRMT1-mediated methylation of 53BP1 promotes DNA binding (not shown), and PRMT5-mediated methylation increases the stability of 53BP1, which contributes to NHEJ repair. BRCA1, a well-established key regulator of HR, is methylated by PRMT1, but its role is unknown. Arginine methylation of RUVBL1 (a cofactor of the TIP60 complex) by PRMT5 facilitates TIP60α-dependent histone H4 Lys16 acetylation (H4K16ac), which blocks 53BP1 recruitment to reinforce HR.

Many studies have revealed that the activity of PRMT5 is crucial in the DDR. PRMT5 methylates three arginine residues (Arg172/174/175) of Rad9^55^. This process is required for the activation of Chk1 signaling and, in turn, S/M and G2/M cell cycle checkpoints. In the DDR, the p53 transcription factor is a major determinant of cell survival or apoptosis^56^. PRMT5 interacts with and methylates p53 at Arg333/335/337 residues, affecting the promoter specificity of p53 associated with apoptosis or cell cycle arrest^57^. Moreover, the translation of p53 is regulated by PRMT5 upon DNA damage via the expression of the translation initiation factor eIF4E^58^. PRMT5-mediated Arg111/113 methylation of E2F1 negatively regulates its protein stability^59^. Under stress caused by DNA damage, E2F1 methylation by PRMT5 is reduced, and consequently, the protein levels of E2F1 are elevated, which contributes to the induction of apoptosis. Upregulation of PRMT5 in cancer downregulates the apoptotic activity of E2F1, contributing to tumorigenesis. Krüppel-like factor 4 (KLF4) is also methylated by PRMT5 at Arg374/376/377 residues. This methylation inhibits VHL-mediated ubiquitination, thereby increasing the protein stability of KLF4^60^. Stress induced by DNA damage increases PRMT5 protein levels and subsequently facilitates the methylation and accumulation of KLF4, modulating the cell cycle and survival of cancer cells. Consistent with these outcomes, aberrant accumulation of PRMT5 and subsequent KLF4 methylation/accumulation correlate with poor prognosis in breast cancer. PRMT5 plays a role in homologous recombination (HR)-mediated DNA repair through arginine methylation of the TIP60 complex^61^. PRMT5 methylates the Arg205 residue of RUVBL1, a cofactor of the TIP60 complex, which promotes TIP60/KAT5-dependent chromatin acetylation and subsequent 53BP1 removal from double-strand break sites. Moreover, the loss of PRMT5 leads to aberrant splicing of DNA repair regulators, including TIP60/KAT5 histone acetyltransferase (HAT) and KMT5C/SUV4-20H2 lysine methyltransferase^62^. A decrease in TIP60α expression by aberrant splicing of TIP60 results in the reduction in TIP60-mediated chromatin acetylation and, in turn, defects in HR. In addition to HR, PRMT5 is implicated in the NHEJ pathway via arginine methylation of 53BP1^63^. Hwang et al. found that the GAR motif of 53BP1 is competitively methylated by PRMT1 and PRMT5. While asymmetric dimethylation of 53BP1 by PRMT1 affects its DNA-binding activity, symmetric dimethylation by PRMT5 regulates its protein stability. Inhibition or deletion of PRMT5 leads to a decrease in 53BP1 protein levels and defects in the NHEJ process (Fig. 4).

## The roles of PRMTs in cancer

With the accumulation of studies on the biological and pathological roles of protein arginine methylation, evidence for a direct link between PRMTs and cancer is emerging. In this section, we discuss the roles of PRMTs in cancer and the molecular mechanisms of each isoform (Table 2).

**Table 2:** The roles of PRMTs in cancer.

PRMTs	Cancer type	Expression	Function	Biological mechanism	Ref.
*PRMT1*					
	Breast cancer	High	Oncogenic	Activation of IGF-1 signaling by ERα methylation in breast cancer	^207^
				EZH2 methylation (R342) leading to an increase in EMT	^65^
				C/EBPα methylation (R35/156/165) leading to activation of *Cyclin D1* expression	^156^
				Activation of ZEB1 transcription leading to cell growth and metastasis	^208^
	Pancreatic cancer	High	Oncogenic	Enhancement of oncogenic GLI1 function by R597 methylation	^67^
				HSP70 methylation leading to stabilization of *BCL2* mRNA	^209^
	Colorectal cancer	High	Oncogenic	Activation of EGFR signaling through EGFR methylation (R198/200)	^66^
	Lung	High	Oncogenic	Regulation of the EMT through Twist1 methylation (Arg34)	^68^
	HCC	High	Oncogenic	Downregulation of *CDKN1A*	^210^
	Melanoma	High	Oncogenic	Increase in ALCAM expression leading to tumor growth and metastasis	^211^
	Head and neck cancer	High	Oncogenic	Increase of growth rate, reduction in migration activity, and increase in *E-cadherin* expression	^212^
	ESCC	High	Oncogenic	Activation of Hedgehog signaling leading to tumor growth, migration, and metastasis	^213^
*PRMT2*					
	Breast cancer	High	Oncogenic	Three spliced variants of PRMT2 are overexpressed in breast cancer; they bind to and activates ERα	^214^
		Low	Tumor suppressive	Downregulates *Cyclin D1* expression	^69^
	Glioblastoma	High	Oncogenic	Transcriptional activation of oncogenes via H3R8me2a	^70^
*PRMT3*					
	Pancreatic cancer	High	Oncogenic	Activation of GAPDH by methylation (R248) and enhancement of glycolysis in cancer	^215^
*CARM1*					
	Breast cancer	High	Oncogenic	Upregulation of *Cyclin E1* leading to the promotion of S-phase entry	^34^
				Enhancement of tumor progression and metastasis through BAF155 methylation (R1064)	^71^
				Stabilization of LSD1 protein by methylation (R838)	^216^
		—	Tumor suppressive	Inhibition of cell proliferation and induction of differentiation in breast cancer	^74^
				Sensitization to chemotherapy drugs through MED12 methylation (R1862/1912)	^75^
	Colorectal cancer	High	Oncogenic	Activation of Wnt/β-catenin transcription and cancer cell growth	^217^
	Pancreatic cancer	Low	Tumor suppressive	Suppression of cell growth and glutamine metabolism through MDH1 methylation (R248)	^218^
	HCC	Low	Tumor suppressive	Inhibition of GAPDH1 by arginine methylation (R234) leading to facilitation of glycolysis in liver cancer cells	^219^
	Ovarian cancer	high	Oncogenic	Promotion of EZH2-mediated silencing of EZH2/BAF155 target tumor suppressor genes	^220^
	AML	high	Oncogenic	Methylation of RUNX1 (R223) by CARM1 blocks myeloid differentiation	^221^
				Facilitation of myeloid leukemogenesis	^222^
*PRMT5*					
	Lymphoma	High	Oncogenic	Activation of WNT/β-catenin and AKT/GSK3β signaling in lymphoma	^223^
	Leukemia/lymphoma	High	Oncogenic	Suppression of the transcription of RB family	^224^
	DLBCL	High	Oncogenic	PRMT5 upregulation by BCR-BKT-NF-κB signaling	^93^
	AML	—	Oncogenic	Regulation of alternative splicing through SRSF1 methylation	^225^
		—	Oncogenic	Silencing of miR-29b and an increase in SP1 and FLT3 expression	^94^
	Breast cancer	High	Oncogenic	Regulation of alternative splicing through ZNF326 methylation (R175)	^188^
				Increase in resistance to chemotherapeutics by regulating stemness-related genes such as OCT4/A, KLF4, and C-Myc	^226^
				Promotion of cell proliferation through interaction with TRAF4 in the nucleus	^227^
				Essential for breast cancer stemness via the activation of *FOXP1* transcription	^79^
	Lung cancer	High	Oncogenic	Repression of miR-99 family transcription and activation of FGFR3/ERK/AKT pathway	^99^
				Promotion of lung cancer cell proliferation through direct interaction with and activation of AKT	^228^
				PRMT5-SHARPIN complex-mediated H3R2me1 activates transcription of metastasis-related genes	^229^
				PRMT5-mediated Enolase-1 methylation (R50me1) enhances localization to the surface membrane	^230^
	Prostate cancer	High	Oncogenic	Activation of AR transcription via H4R3me2s with pICln coactivator	^98^
				Methylation of AR (R761), leading to attenuation of AR-mediated transcription involved in differentiation	^231^
	Gastric cancer	High	Oncogenic	PRMT5 expression positively correlates with the expression of GENMIN2, STAT3, and TGFB3, and malignant phenotype	^86^
				Direct interaction with c-Myc to suppress the transcription of *PTEN*, *CDKN2C*, *CDKN1A*, *CDKN1C*, and *p63*	^232^
				PRMT5-mediated histone methylation recruits DNMT3A to silence *IRX1*	^85^
	HCC	High	Oncogenic	Enhancement of invasive activity via regulation of MMP-2 expression	^87^
				Promotion of HCC proliferation by downregulating BTG2 expression	^88^
	Pancreatic cancer	high	Oncogenic	Downregulation of FBW7 leading to stabilization of c-Myc	^89^
				Activation of EGFR-AKT-GSK3β-β-catenin signaling leading to cell growth	^90^
	Colorectal cancer	High	Oncogenic	Methylation YBX1 (R205) is essential for NF-κB activation and CRC growth and migration	^84^
	Melanoma	High	Oncogenic	SHARPIN facilitates PRMT5 activity that increases SOX10 and PAX3 expression	^95^
				Regulation of *MDM4* expression via alternative splicing, which results in resistance to the CDK4/6 inhibitor	^233^
	Glioblastoma	High	Oncogenic	Silencing of the *ST7* tumor suppressor gene leading to tumor cell growth and survival	^96^
	Bladder cancer	High	Oncogenic	Enhancement of NF-κB activation, thereby increasing BCL-XL/cIAP1	^92^
	MTAP deleted cancer			Increased endogenous MTA inhibits PRMT5 activity and induces vulnerability toward PRMT5	^101–103^
*PRMT6*					
	Gastric cancer	High	Oncogenic	Enhances global H3R2me2a and suppresses several tumor suppressor genes including *PCDH7*, *SCD*, and *IGFBP5*	^234^
	Endometrial cancer	High	Oncogenic	Facilitation of EMC cell proliferation and migration via the activation of AKT/mTOR signaling	^235^
	Lung cancer	High	Oncogenic	Activation of tumor-associated macrophages via interaction with ILF2	^236^
	HCC	Low	Tumor suppressive	Methylation of CRAF (R100) by PRMT6 inhibits RAS/RAF binding and MEK-ERK signaling	^202^
*PRMT7*					
	Breast cancer	High	Oncogenic	Increase in MMP9 expression	^237^
				Promotion of metastasis through SHANK2 methylation (R240)-mediated FAK activation	^106^
	Lung (NSCLC)	High	Oncogenic	Promotion of the invasion and colony formation through interaction with HSPA5 and EEF2	^238^
	Renal cell carcinoma	High	Oncogenic	Upregulation of c-Myc expression via β-catenin methylation	^239^
*PRMT9*					
	HCC	High	Oncogenic	Promotion of invasion and metastasis through PI3K/AKT/GSK3β/Snail signaling activation	^240^

*HCC* hepatocarcinoma, *ESCC* esophageal squamous-cell carcinoma, *AML* acute myeloid leukemia, *DLBCL* diffuse large B-cell lymphoma, *MTAP* methylthioadenosine phosphorylase, *NSCLC* non-small cell lung carcinoma.

### Type I PRMTs

#### PRMT1

PRMT1 is the most predominant enzyme in the PRMT family, and its activity is responsible for more than 90% of the overall arginine methylation in mammalian cells^64^. The dysregulation of PRMT1 expression and its pathological mechanisms in various human carcinomas are summarized in Table 2. For instance, EZH2 (enhancer of zeste homolog 2) is asymmetrically dimethylated at Arg342 by PRMT1^65^, which leads to an increase in EZH2 levels because TNF receptor associated factor 6 (TRAF6)-mediated ubiquitination is interrupted. Upregulation of EZH2 by Arg342 methylation consequently reduces the expression of EZH2 target genes such as *HOXA10*, *DAB2IP*, *HOXA9*, and *HOXA7*, promoting breast cancer cell migration and metastasis. Indeed, the expression levels of PRMT1 and the methylation levels of the Arg342 residue of EZH2 correlate with poor clinical outcomes in breast cancer patients, suggesting the utility of PRMT1 as a diagnostic marker and therapeutic target for cancer. In colorectal cancer patients, PRMT1-mediated Arg198/200 methylation of EGFR is correlated with tumor growth, a high recurrence rate after cetuximab treatment, and reduced overall survival^66^. PRMT1 methylates GLI1 at the Arg597 residue, which enhances its transcriptional activity^67^. In pancreatic ductal adenocarcinoma, increased PRMT1 expression correlates with GLI1 expression and leads to SMO-independent GLI1 activation, thereby mediating its oncogenic functions. PRMT1-mediated Twist1 methylation is involved in the regulation of the epithelial-mesenchymal transition (EMT) in lung cancer cells^68^. The Twist1 transcription factor, known as an E-cadherin repressor, is methylated by PRMT1 at the Arg34 residue to promote its repressive activity. Upregulation of PRMT1 expression in lung cancer is linked to a decrease in E-cadherin and an increase in N-cadherin levels, which stimulates cell migration, invasion, and metastasis.

#### PRMT2

The role of PRMT2 in cancer remains controversial. In breast cancer cells, PRMT2 is recruited to the AP-1-binding site of the *CCND1* promoter and ERα binding is simultaneously suppressed^69^. Depletion of PRMT2 expression leads to an increase in estrogen-induced *CCND1* expression and promotion of cell proliferation and colony formation, indicating That PRMT2 has tumor-suppressive activity. In contrast, an oncogenic function of PRMT2 in glioblastoma has been reported^70^. PRMT2 expression is elevated in glioblastoma and is correlated with tumor grade. The PRMT2-mediated H3R8me2a modification is implicated in the activation of the oncogenic transcriptome, leading to the enhancement of GBM cell growth and tumorigenesis.

#### CARM1

The role of CARM1 in cancer is still debated. CARM1 positively regulates the transcription of *CCNE1* via H3R17 and H3R26 methylation in collaboration with E2Fs and ACTR^34^. In high-grade breast tumors, the mRNA levels of *CARM1* and *ACTR* are elevated, indicating an oncogenic role of CARM1 in breast cancer. CARM1-mediated BAF155 methylation promotes cancer cell migration and metastasis^71^. The chromatin remodeling factor BAF155 (BRG1-associated factor 155) is methylated by CARM1 at Arg1604, which modulates the chromatin association patterns of BAF155. Arg1604 methylation of BAF155 facilitates cell migration and metastasis and correlates with breast cancer progression, malignancy, and recurrence-free survival. Arginine methylation of pyruvate kinase 2 (PKM2) by CARM1 is implicated in tumorigenesis via modulation of energy metabolism^72^. The Arg445/447/455 residues of PKM2 are methylated by CARM1. This methylation does not affect PKM2 enzymatic activity and is involved in regulating mitochondrial respiration in cancer cells. PKM2 methylation leads to decreased Ca^2+^ uptake and diminished mitochondrial membrane potential, causing an increase in cell proliferation, migration, and metastasis. Recently, the oncogenic function of CARM1 in *CBP/P300*-mutated lymphomas was well characterized^73^. Inhibition of CARM1 activity slows diffuse large B-cell lymphoma (DLBCL) growth, which is positively correlated with *CBP/P300*-mutation status, indicating that the *CBP/P300* mutation in cancer creates a vulnerability to targeting CARM1 activity.

In contrast to these oncogenic functions, several reports have described the role of CARM1 as a tumor suppressor. As a coactivator of the estrogen receptor ERα, CARM1 regulates estrogen-dependent breast cancer cell proliferation and differentiation^74^. CARM1 suppresses estradiol (E2)-dependent cell cycle progression and proliferation of breast cancer cells via modulation of the ERα-mediated transcription of proteins, especially *p21*^*WAF1*^, *p27*^*KIP*1^, *Cyclin G2*, *MAZ*, *KRTAP10.12*, and *GATA-3*. In ER-positive breast cancers, the expression level of CARM1 is positively correlated with ERα levels and inversely correlated with tumor grade, suggesting that CARM1 is a biomarker of well-differentiated breast cancer cells. In addition, CARM1 activity contributes to the sensitization of cancer cells to chemotherapy drugs via arginine methylation of RNA polymerase II mediator complex subunit 12 (MED12)^75^. CARM1 interacts with and methylates MED12 at Arg1862/1912 located in the C-terminal proline-glutamine-leucine-rich (PQL) domain. ChIP-seq analysis revealed that arginine methylation of MED12 enhances chromatin association with target genes, especially *p21*^*WAF1*^, resulting in suppression of *p21*^*WAF1*^ transcription. Methylation of MED12 renders cancer cells sensitive to chemotherapy drugs under in vitro and in vivo conditions, and higher levels of MED12 and CARM1 correlate with a better response to chemotherapy drugs.

#### PRMT6

PRMT6 demonstrates oncogenic activity by inducing the addition of the epigenetic repressive H3R2me2a mark on tumor suppressor genes, such as *p21*^*WAF1*^ and *p16*^*INK4A*^, which facilitates cell proliferation and prevents senescence^76^. In addition, PRMT6-mediated H3R2me2a impedes the recruitment of UHRF1 (an accessory factor of DNMT1) onto chromatin, leading to DNA hypomethylation^77^. Indeed, PRMT6 expression inversely correlates with global DNA methylation in many human cancer cells, and PRMT6 depletion or inhibition restores DNA methylation. These observations demonstrate the potential of targeting PRMT6 for cancer therapy.

### Type II PRMT

#### PRMT5

A major type II enzyme, PRMT5, is emerging as the most promising target for a range of solid and blood cancers. Overexpression or dysregulation of PRMT5 has been observed in various cancer types, including breast^78,79^, lung^80,81^, ovarian^82^, prostate^83^, colorectal^84^, gastric^85,86^, liver^87,88^, pancreatic^89,90^, head and neck^91^, bladder^92^, lymphoma^93,94^, melanoma^95^, and glioma^96^. Epigenetically, PRMT5 associates with BRG1- and hBRM-based hSWI/SNF chromatin remodeling complexes and induces H3R8me2s and H4R3me2s modifications, repressing the transcription of tumor suppressor genes such as suppressor of tumorigenicity 7 (*ST7*) and nonmetastatic 23 (*NM23*)^97^. The PRMT5-pICln (but not the MEP50) complex is recruited to the proximal region of the androgen receptor (*AR*) promoter and mediates symmetric dimethylation of H4R3, which acts as an epigenetic activation modification^98^. An increase in AR expression mediated by PRMT5 promotes the growth of castration-resistant prostate cancer cells. The epigenetic regulation of cancer-specific miRNA expression by PRMT5 is critical for tumor growth, progression, and metastasis. The PRMT5-mediated H4R3me2s modification silences miR-29b expression, resulting in increased levels of Sp1 and FLT3. This increase leads to the growth of cancer cells in acute myeloid leukemia^94^. In addition, overexpression of PRMT5 in lung cancer enriches the epigenetic repressive mark H4R3me2s on the promoter of the miR-99 family and subsequently suppresses the expression of member miRs^99^. Reduced expression of miR-99 family members increases the expression of fibroblast growth factor receptor 3 (FGFR3) and, in turn, activates ERK1/2 and AKT signaling, promoting lung cancer cell migration and invasion. PRMT5 also contributes to carcinogenesis via the arginine methylation of several oncoproteins and tumor suppressors (Table 2). For instance, programmed cell death 4 (PDCD4), a tumor suppressor, is methylated at Arg110 by PRMT5^78^. High expression of PDCD4 alone correlates with better outcomes for breast cancer patients. However, patients with both high PDCD4 and PRMT5 demonstrate poor prognoses, suggesting that arginine methylation of PDCD4 by PRMT5 decreases the ability of PDCD4 to suppress cancer cell growth. Indeed, Arg110 methylation of PDCD4 by PRMT5 modulates PDCD4 subcellular translocalization from the nucleus to the cytoplasm and facilitates its interaction with eIF4A in the cytoplasm, leading to enhanced cancer cell viability^100^.

Recently, several reports have been published on the correlation between S-methyl-5′-thioadenosine phosphorylase (*MTAP*) gene deletion and susceptibility to PRMT5 action, which is worth considering^101–103^. The chromosome 9p21 (chr9p21) locus, which encodes the *CDKN2A* gene, is homozygously deleted in approximately 15% of all human cancers, with frequent codeletion of the *MTAP* gene, in 80–90% of tumors, along with *CDKN2A* deletion. Due to the intracellular accumulation of methylthioadenosine (MTA), an endogenous PRMT5 antagonist, *MTAP* deletion renders cancer cells sensitive to PRMT5. Based on this rationale, the combination of a PRMT1 inhibitor and PRMT5 inhibitor synergistically inhibits the proliferation of cancer cells with *MTAP* deletion^104^.

### Type III PRMT

#### PRMT7

PRMT7 is prominently overexpressed in malignant breast tumors and is associated with the EMT^105^. In PRMT7-overexpressing cells, enriched H4R3me2s at the *E-cadherin* promoter antagonizes the H3K4me3 epigenetic modification and, in turn, represses the transcription of *E-cadherin* during the EMT. The recruitment of PRMT7 to the *E-cadherin* promoter depends on the YY1 transcription factor, and the PRMT7-YY1-HDAC3 ternary complex acts as a transcriptional repressor of *E-cadherin*. In the EMT, SHANK2 (scaffolding protein SH3 and multiple ankyrin repeat domain 2) is symmetrically dimethylated at Arg240 by PRMT7, which activates endosomal FAK/cortactin signaling, contributing to cancer cell invasion, metastasis, and malignancy^106^. As mentioned above, since PRMT7 is a type III PRMT that can only deposit MMA, it is unclear how the SDMA mark is enriched. Perhaps the following possibilities should be considered: the H4R3me1 mark becomes a substrate for PRMT5, or there is an auxiliary factor that can convert PRMT7 into a type II enzyme.

## PRMT inhibitors constitute a novel class of anticancer drugs

As described above, PRMTs regulate various cellular processes, including transcription, mRNA splicing, translation, DNA damage/repair response, and the cell cycle. Since they are closely associated with cancer and tumorigenesis, PRMTs have recently emerged as molecular targets for anticancer drug development and play essential roles in cancer research^17,107^. As a result, enormous efforts have been undertaken to develop effective and selective PRMT inhibitors. Although many candidates are still in the preclinical stage, some inhibitors have entered clinical trials. In Table 3, we summarize the main features of the inhibitors that have been developed thus far and briefly discuss them below.

**Table 3:**
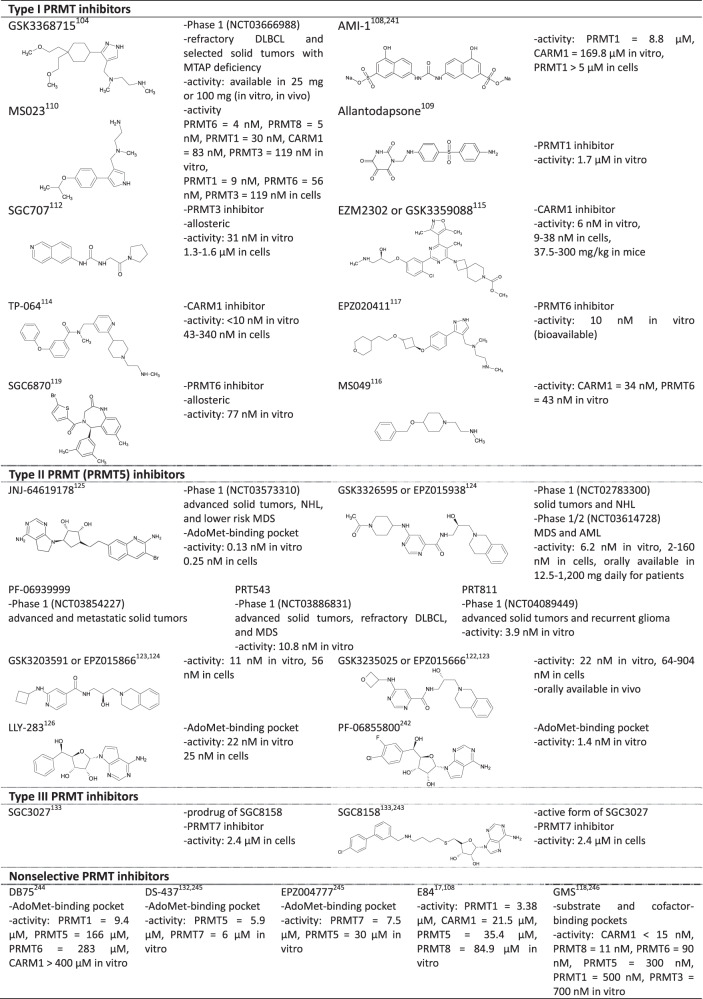
PRMT Inhibitors^241–246^.

### Type I PRMT inhibitors

Most PRMT inhibitors target type I not type II PRMTs. The first discovered PRMT inhibitor was AMI-1 (2004), which inhibits type I PRMTs^108^. Despite its usefulness, more specific and potent inhibitors needed to be developed. This requirement was partially fulfilled by the development of allantodapsone with specificity for PRMT1^109^. MS023 also contributed to the available potent inhibitors by inhibiting type I PRMT at concentrations much lower than those of AMI-1^110^. The in vitro working concentration was reduced from the micromolar to nanomolar range. Finally, GSK3368715 was developed and entered a phase 1 clinical trial in 2018^104^. GSK3368715 is being examined for its use as a treatment for refractory diffuse large B-cell lymphoma and select solid tumors with MTAP deficiency (http://clinicaltrials.gov/ct2/show/NCT03666988). Since the loss of MTAP leads to the accumulation of MTA, an endogenous PRMT5 inhibitor, GSK3368715 can be effective in MTAP-null cancer cells by mediating the blockage of the compensatory relationship between ADMA and SDMA^107^. This finding also suggests that combination therapy with type I PRMT inhibitors and PRMT5 inhibitors may demonstrate synergistic effects^111^.

In contrast to PRMT1 inhibitors that bind mainly to the substrate-binding pocket, a substrate and AdoMet noncompetitive inhibitor, SGC707, has been developed;^112^ this is the first allosteric PRMT3 inhibitor to have been developed. Although SGC707 is insufficient for therapeutic purposes, it is a good reference to encourage the development of other allosteric inhibitors^113^. As efforts to develop type I PRMT inhibitors continued, several CARM1 inhibitors, such as EZM2302 and TP-064, were also identified 2^114,115^. They have demonstrated remarkable efficacy under in vivo and in vitro conditions. MS049 is a dual inhibitor of CARM1 and PRMT6^116^. EPZ020411 is a representative PRMT6 inhibitor that can also inhibit PRMT1, PRMT8, and other PRMTs but has a high affinity for PRMT6^117^. Similarly, many compounds have been developed to inhibit PRMT6, such as GMS, which have an effect in the nanomolar range but lack selectivity^118^. Recently developed SGC6870 is a highly selective inhibitor of PRMT6^119^.

### Type II PRMT inhibitors

Despite many expectations for and investments into the development of type I PRMT inhibitors, only a few satisfactory outcomes have been observed. However, the development of PRMT5 inhibitors has been more successful. This outcome is not surprising, as PRMT5 plays an essential role in cancer stem cell survival, mRNA splicing, and DNA repair processes^61,120,121^. Thus, PRMT5 inhibitors can be useful for treating cancer in mono- or combination therapy with DNA-damaging agents. EPZ015666 was the first PRMT5 inhibitor;^122^ similar compounds have also been developed^123,124^. Among these inhibitors, GSK3326595 has entered phase 1/2 clinical trials (http://clinicaltrials.gov/ct2/show/NCT02783300 and http://clinicaltrials.gov/ct2/show/NCT03614728). These inhibitors bind at the substrate-binding pocket. This binding is enhanced via AdoMet, which competes with MTA^101^. Therefore, EPZ015666 is less effective in MTAP-null cancer cells. Hence, PRMT5 inhibitors that bind at the AdoMet-binding pocket have been developed. LLY-283 and JNJ-64619178 are representative examples^125,126^. Specifically, JNJ-64619178 has entered Phase 1 clinical trials and is being examined for use in the treatment of advanced solid tumors, non-Hodgkin lymphoma, and lower-risk myelodysplastic syndromes (http://clinicaltrials.gov/ct2/show/NCT03573310). In 2019, PF-06939999 (http://clinicaltrials.gov/ct2/show/NCT0385427), PRT543 (http://clinicaltrials.gov/ct2/show/NCT03886831), and PRT811 (http://clinicaltrials.gov/ct2/show/NCT04089449) also entered phase 1 clinical trials.

Interest in PRMT5 inhibitors has been increasing, for several reasons for this: PRMT5 inhibitors have successfully entered clinical trials, and the relationship between *MTAP* loss and PRMT5 activity has been demonstrated^103^. PRMT5 has a unique characteristic that requires MEP50 to serve as its complex partner^127^. Considering these observations, scientists of recent studies have suggested the development of allosteric PRMT5 inhibitors that stabilize MTA or enhance the formation of the PRMT5-MTA complex and inhibitors that disrupt the formation of the PRMT5-MEP50 complex^3^. These suggestion are interesting. MS4322, the first developed PRMT5 degrader, is an example of the application of proteolysis targeting chimera (PROTAC)^128^. PROTAC is a technology employed to degrade a specific target protein in a proteasome-dependent manner by recruiting the E3 ubiquitin ligase^129^. Since it demonstrates broad applications and has enabled the resistance to small-molecule inhibitors to be overcome, PROTAC technology has been frequently used for novel drug discovery and development^130^. MS4322 forms a link between the structure of EPZ015666 and the von Hippel-Lindau E3 ubiquitin ligase ligand, and hence, MS4322 effectively and selectively inhibits PRMT5. This discovery is meaningful, as it confirmed the possibility that a therapeutic PRMT degrader can be developed.

### Type III PRMT inhibitors

PRMT7 was identified in 2004 and is associated with metastasis and DNA damage^131^. Although PRMT7 is considered a potential target for treating breast cancer^105^, studies on the development of PRMT7 inhibitors are still limited. DS-437 was developed as a dual inhibitor of PRMT5 and PRMT7^132^. Recently, SGC3027 was developed as the first PRMT7 inhibitor^133^. It is a prodrug that can be converted to the active form: SGC8158.

## Future perspectives

Protein arginine methylation, as reviewed in this paper, plays an essential role in maintaining biological homeostasis^4^. Dysregulation of arginine methylation is observed not only in cancer cells but also in various tumors (Table 2). Hence, the development of anticancer drugs targeting PRMTs has gained traction (Table 3). The fact that PRMT inhibitors are included in multiple clinical trials may be sufficient to fuel research examining arginine methylation. However, several issues still need to be addressed to better understand the roles of arginine methylation and successfully develop its inhibitor: (1) novel PRMT substrates need to be identified and characterized, (2) a regulatory mechanism for arginine methylation needs to be found, and (3) isoform-specific inhibitors need to be developed.

PRMTs govern diverse cellular processes, including transcription, signaling pathways, splicing, cell cycle progression, and DNA damage and repair processes, via methylation of a variety of substrate proteins, as summarized in Table 1. Research on arginine methylation is still in the early stages. Many proteins can be methylated at arginine residues, and this modification regulates various cellular responses. Therefore, one of the obvious future goals is to clarify the downstream pathways by identifying novel substrates of PRMTs, which will guide help us to understand the mechanisms of various diseases, including cancer, and establish treatment strategies. Next, the regulatory mechanism of arginine methylation should be more clearly understood. In contrast to other PTMs, arginine methylation is known to be quite stable^2,5^. Arginine methylation appears to be a nondynamic and static reaction, as the presence of dedicated arginine demethylase that enables cycles of methylation and demethylation has not been identified. As several reports have shown that the levels of arginine methylation change dynamically depending on the cellular environment^134,135^, we are confident that a arginine demethylase will soon be discovered. In addition, the regulatory mechanisms of PRMT activity in cellular systems are poorly understood. PRMT5 is active only when it interacts with MEP50^136,137^. In some cases, the enzyme activity of PRMTs can be regulated by other PTMs, such as phosphorylation. PRMT5 can be phosphorylated at tyrosine residues by JAK2-V617F or Src family kinases, which leads to a decrease in its methyltransferase activity^63,138^. In contrast, the phosphorylation of PRMT5 Thr80 by RhoA-activated kinase increases its methyltransferase activity^139^. However, since these regulatory mechanisms are observed under limited and special circumstances, the detailed regulatory mechanism of arginine methylation is a concept that must be explored. Finally, developing PRMT inhibitors as novel anticancer drugs requires a careful approach. PRMTs, especially PRMT1 and PRMT5, are considered promising targets for the development of anticancer drugs because of their pro-oncogenic functions^2,3,18^. However, since arginine methylation is an essential response to normal cell growth and homeostasis maintenance^3–5^, nonselective and indiscriminate inhibition of PRMTs is likely to lead to undesirable effects. Therefore, it is necessary to select and target tumors that are relatively vulnerable to PRMT inhibition. From this perspective, it is quite remarkable that the lack of the *MTAP* gene increases sensitivity to PRMT5 inhibitors^101–104^ and that *CBP/P300* mutation creates vulnerability to CARM1 inhibitors^73^. In addition, the development of isoform-specific inhibitors will ensure successful cancer treatment.

In summary, systematic approaches to arginine methylation, including the issues discussed above, will not only provide a better understanding of biological phenomena but also lead to the development of a novel class of anticancer drugs.
